# Left atrial late gadolinium enhancement and mitral regurgitation in subjects with atrial fibrillation

**DOI:** 10.1186/1532-429X-17-S1-P358

**Published:** 2015-02-03

**Authors:** Karl Grunseich, Dana C Peters, Albert J Sinusas, Hamid Mojibian, Mark Marieb, Daniel Cornfeld, Lauren A Simprini

**Affiliations:** 1 Yale University School of Medicine, New Haven, CT, USA

## Background

Late gadolinium enhancement (LGE) is used to evaluate fibrosis of the left atrium (LA) in subjects with atrial fibrillation (AF). LA fibrosis by LGE has been shown to correlate with LA volumes. However, within AF subjects, the association of mitral regurgitation (MR) with LA LGE is not well described. Pathological studies have demonstrated atrial fibrosis and tissue disorganization in cardiac diseases, including valvular disease. The current study evaluates the relationship between LA LGE, LA volumes, and MR in subjects with AF.

## Methods

A retrospective chart review of AF subjects imaged with the whole heart 3D LGE MRI sequence at our institution (Siemens 1.5T) from 2012-2013 identified 22 cases. 4 subjects were excluded due to either prior ablation procedure (3) or inadequate images for analysis (1). The presence of visually detected MR was identified by clinical MRI reports. LGE in the LA was scored using a semi-quantitative approach based on its visually-assessed presence in 18 locations. Measurements of LA volumes were approximated by biplane area-length method at atrial end diastole. Left ventricular stroke volumes were quantitated via endocardial tracings of steady state free precession short axis stack cine images at left ventricular end diastole and end systole using semi automated threshold detection, excluding trabeculations and papillary muscles from left ventricular volumes (Circle Cardiovascular Imaging software v4.2). Aortic forward flow by phase contrast imaging was subtracted from left ventricular stroke volume to determine mitral regurgitant fraction (MRF). Regressions and t-tests were performed using JMP 10 (SAS Institute Inc. Cary NC).

## Results

**Figure 1:**
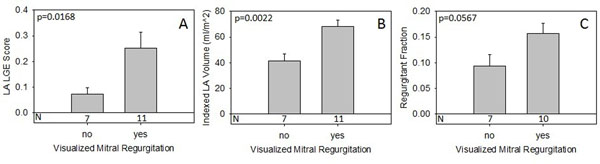
**Visually detected mitral regurgitation is associated with higher LGE score (A) and LA volume (B) with a trend toward higher quantitative regurgitant fraction (C).** Values are mean +S.E. One patient lacked phase contrast imaging necessary for calculating RF.

**Figure 2:**
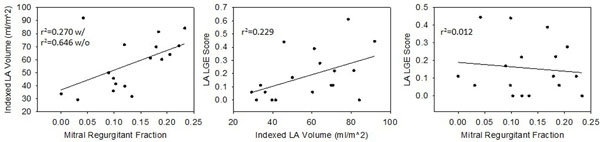
**Relationships between mitral regurgitant fraction, LA volume, and LA LGE Score.** In left panel, r^2^ is reported with and without inclusion of one bivariate outlier.

Subject (N=18) characteristics were 83% male, mean age 55.2±10.6, mean BMI 29.5±5.3, 67% hypertensive, 11.2% congestive heart failure, and 5.6% diabetic. MR was visualized in 61% of subjects (8 mild, 2 moderate, 1 not described). The presence of MR was significantly associated with a higher LA LGE score (p=0.017) and increased LA volume (p=0.002), with a trend toward increased quantitative MRF (p=0.057; Figure 1). LA volume was associated with MRF (r^2^=0.65, p<0.001) and with LA LGE (r^2^=0.23, p=0.045; Figure 2). Direct association between MRF and LA LGE (r^2^=0.01 p=0.670) was not observed.

## Conclusions

Significant MR has the potential to increase LA volume and result in atrial remodelling. Among AF subjects, we demonstrated that the presence of MR was significantly associated with LA LGE score and LA volume. We have also demonstrated an association between LA volumes and LA LGE. Direct association between MRF and LA LGE was not observed, possibly due to a relatively small sample size and number of subjects with significant regurgitant fractions. Future study of a larger cohort, including subjects without AF and with a wider range of MR, may be of utility to further investigate this relationship.

## Funding

This work was partly funded by NHLBI R21 HL 098573.

